# An interesting case of pacemaker endocarditis

**DOI:** 10.1007/s12471-019-01310-2

**Published:** 2019-07-25

**Authors:** K. K. Sahu, A. K. Mishra, A. A. Sherif, A. Doshi, B. Koirala

**Affiliations:** 1 Department of Internal Medicine, Saint Vincent Hospital, Worcester, Massachusetts United States; 2 Department of Cardiovascular diseases, Saint Vincent Hospital, Worcester, Massachusetts United States; 3 Department of Internal Medicine, Reliant Medical Group, Saint Vincent Hospital, Worcester, Massachusetts United States

## Answer

**Fig. 1:**
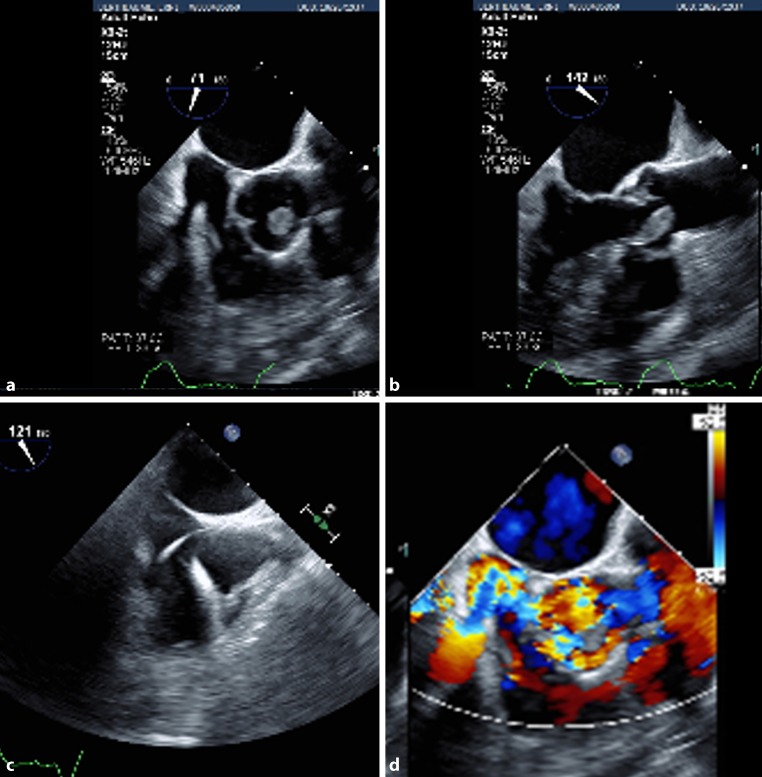
**a** Short axis view and **b** long axis view at the level of mid oesophagus showing 1.5 × 1.4 cm echo-dense structure on the right coronary cusps of the aortic valve. Upper oesophageal view **c** showing right atrial pacer lead with thin filamentous 1‑cm long freely mobile echo-dense structure and **d** Doppler showing severe aortic regurgitation

We changed antibiotics to vancomycin and cefazolin suspecting it to be *Staphylococcus aureus* bacteraemia. On follow-up, the sputum culture grew *Staphylococcus aureus* and the blood culture grew *Aerococcus urinae*. Source of bacteraemia was not clear as his urine culture was positive for *Pseudomonas aeruginosa*, which was likely due to colonisation. Suspicion of infective endocarditis (IE) remained high and was evaluated with transoesophageal echocardiography (TEE) which showed a right atrial pacer lead vegetation (1 × 1 cm) and an another 1.5 × 1.4 cm echo-dense structure on the right coronary cusps of aortic valve (Fig. 1b, d). Patient was continued on antibiotics, unfortunately he succumbed to illness before he could be taken up for aortic valve replacement and pacemaker removal. As per modified Duke’s criteria, our patient qualified for IE (1 major criterion: positive findings in TEE, 3 minor criteria: predisposing heart conditions, temperature >38.0 °C (100.4 °F), microbiological evidence of positive blood culture, but does not meet a major criterion).

*Aerococcus* is a Gram-positive, alpha haemolytic, catalase-negative cocci which is a rare cause of urinary tract infection in elderly. First reported in 1989, it is now known to cause clinically significant infections, such as soft tissue infections, urinary tract infections, septicaemia, endocarditis. Recently, Yabes et al. did a review of 43 cases of *Aerococcus urinae*-associated infective endocarditis [1]. They found that only 29 of these cases had documented urinary tract-related pathologies or procedures (including cystoscopy, urethral stricture, BPH, indwelling catheter). Similarly, in the study by Christensen et al., the urinary tract system was considered as the focus of infection in 16 out of 17 cases, however, *Aerococcus* was isolated from the urine of only nine patients [2]. Recently, many newer laboratory and imaging studies have improved the detection of rare cardiac myocardial and valvular pathologies [3–6, 7].

*Aerococcus *in Gram stain classically has tetrad morphology, but can also appear in clusters and irregular pairs and can be, at times, confused with *Staphylococcus*. Catalase negativity can be helpful in differentiating these two morphologically similar species. Also, to note that, in the blood agar, *Aerococcus* usually displays alpha haemolysis (resembling streptococci). 16S rRNA gene sequencing and matrix-assisted laser desorption ionisation time of flight mass spectrometry (MALDI-TOF-MS) are increasingly being used for *Aerococcus* identification [8]. Unfortunately, due to the rarity, we lack controlled scientific trials and formalised guidelines. In most cases, therapy is often empirical and based on expert opinion.
